# Supplementary figures and images for: Aging-regulated TUG1 is dispensable for endothelial cell function
Source: PLoS One. 2022 Sep 29;17(9):e0265160. doi: 10.1371/journal.pone.0265160 (PMC9522302; doi:10.1371/journal.pone.0265160)

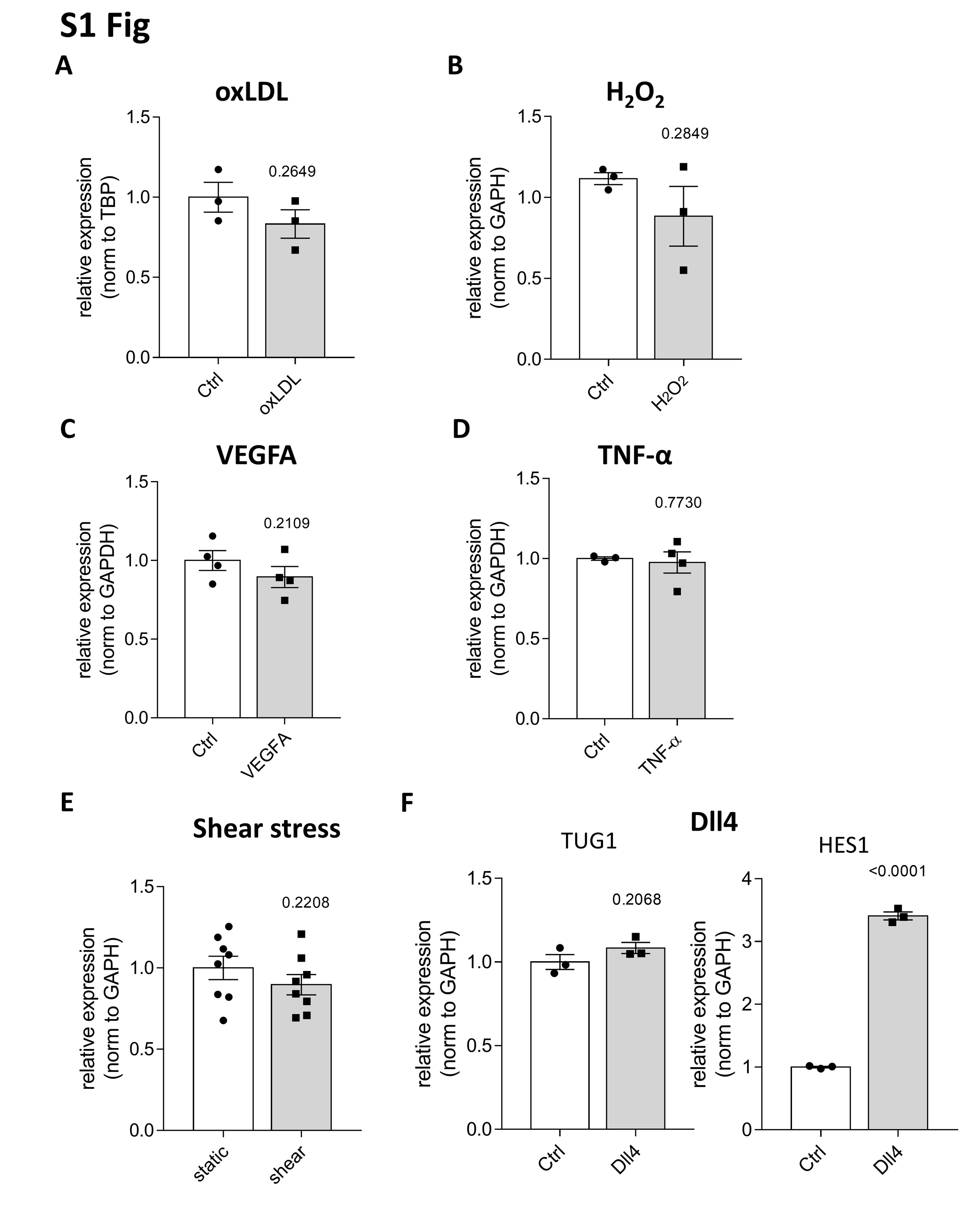

Supplement: S1 Fig — TUG1 RNA levels were measured by RT-qPCR after stimulation with (A) 50 μg/ml oxLDL for 48h (n = 3; SEM; unpaired t-test), (B) 200 μM H2O2 for 1h (n = 3; SEM; unpaired t-test), (C) 50 ng/ml VEGFA for 24h (n = 4; SEM; paired t-test), (D) TNFa 10 ng/ml for 24h (n = 3–4; SEM; unpaired t-test), (E) shear stress with 20 Dyn/cm2 for 72h (n = 8; SEM; paired t-test; cells treated for the same time under static conditions were taken along as Ctrl) and (F) 1 μg/ml rDll4 for 24h (n = 3; SEM; unpaired t-test; Hes Family BHLH Transcription Factor 1 (HES1) served as a Ctrl). Expression is normalized to GAPDH or TBP as determined by RT-qPCR. (TIF) [file pone.0265160.s001.tif]

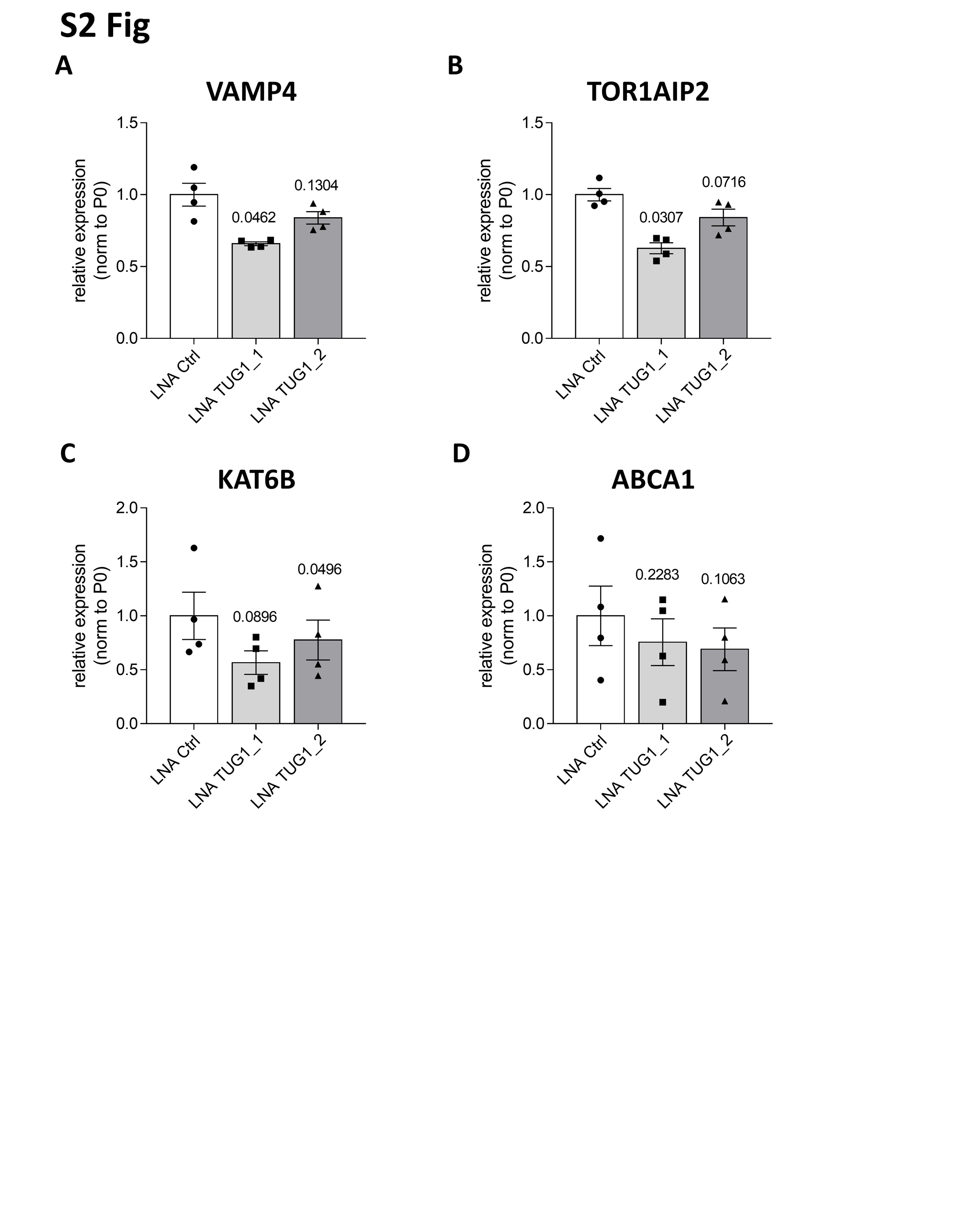

Supplement: S2 Fig — HUVECs were transfected with two LNA GapmeRs against TUG1—LNA TUG1_1 and LNA TUG1_2 –and LNA Ctrl and expression levels of (A) VAMP4, (B) TOR1AIP2, (C) KAT6B and (D) ABCA1 were measured after 48 hours by RT-qPCR. Expression is relative to P0 (n = 4; SEM; RM one-way ANOVA with Greenhouse-Geisser correction and Holm-Sidak multiple comparison test). (TIF) [file pone.0265160.s002.tif]

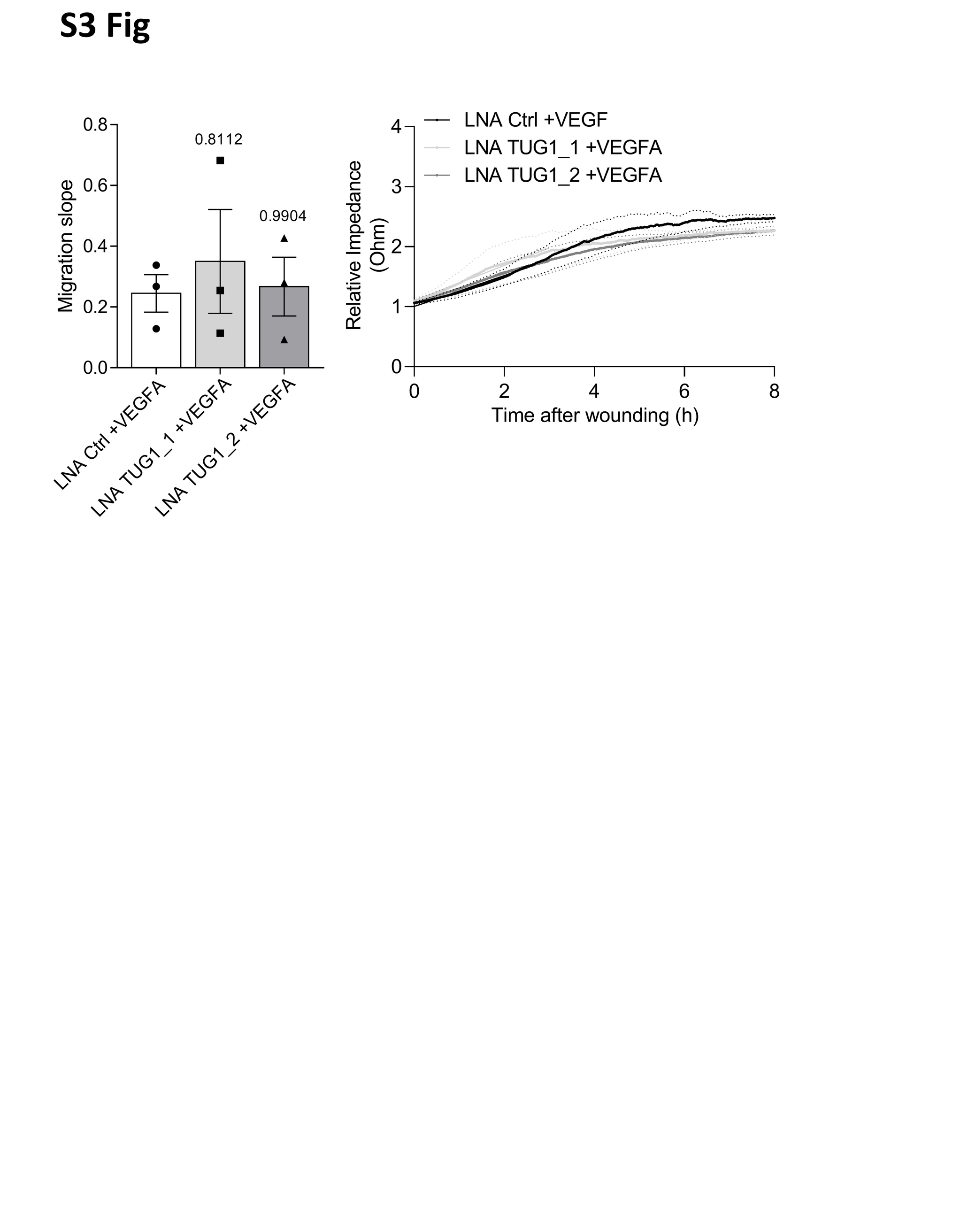

Supplement: S3 Fig — A confluent monolayer of transfected HUVECs (LNA TUG1_1, LNA TUG1_2 or LNA Ctrl) were wounded in an ECIS setup and reestablishment was analyzed (n = 3; SEM; RM one-way ANOVA with Greenhouse-Geisser correction and Holm-Sidak multiple comparison test). (TIF) [file pone.0265160.s003.tif]

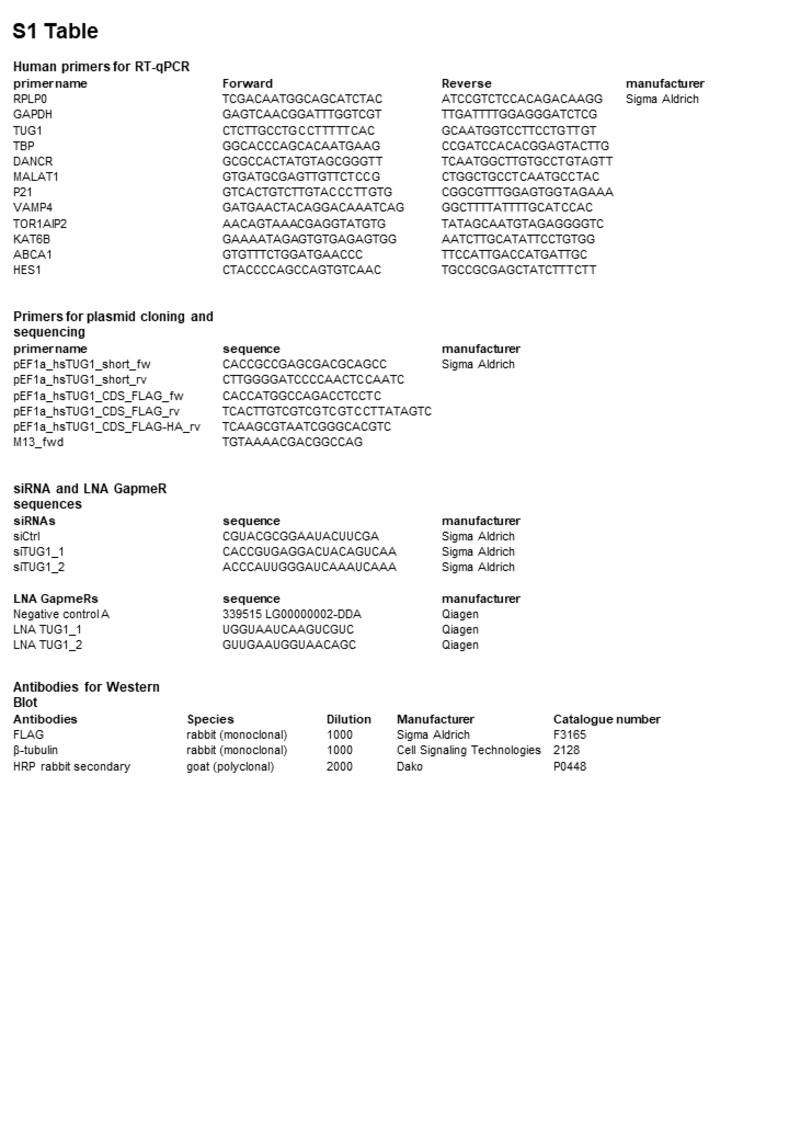

Supplement: S1 Table — Oligonucleotide sequences used for RT-qPCR, as well as for cloning and sequencing are listed. Sequences that were used to synthesize siRNAs and LNA gapmers can be found here. There is also a list of the antibodies and their dilutions used to generate western blots. (TIF) [file pone.0265160.s005.tif]
