# Supplementary material for: Aging-regulated TUG1 is dispensable for endothelial cell function
Source: PLoS One. 2022 Sep 29;17(9):e0265160. doi: 10.1371/journal.pone.0265160 (PMC9522302; doi:10.1371/journal.pone.0265160)
Supplement: S1 Raw image — Uncropped images of the western blots used in Fig 4. (PDF) [file pone.0265160.s004.pdf]

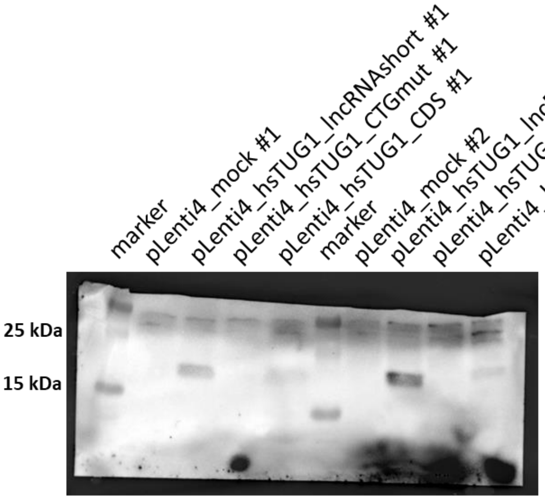

FLAG

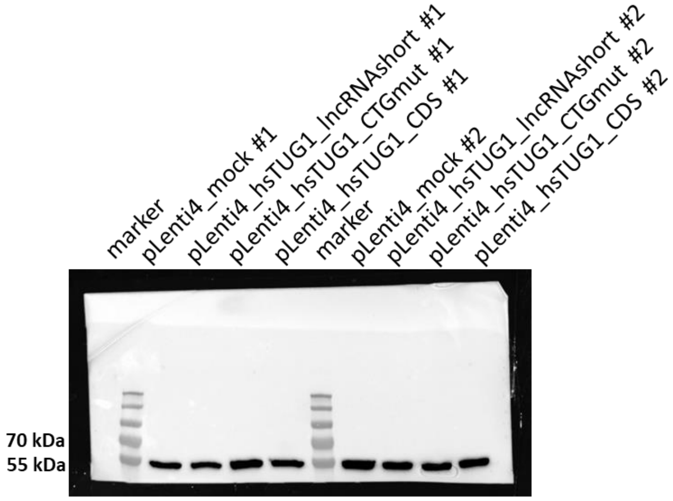

β-Tubulin

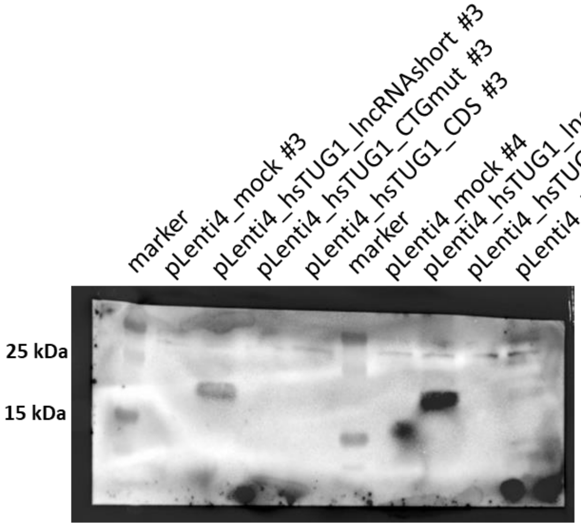

FLAG

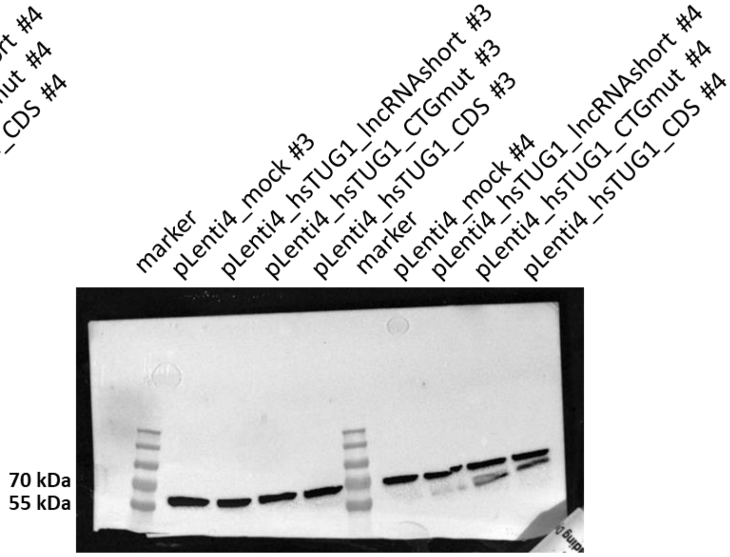

β-Tubulin
