# Supplementary material for: Aging-regulated TUG1 is dispensable for endothelial cell function
Source: PLoS One. 2022 Sep 29;17(9):e0265160. doi: 10.1371/journal.pone.0265160 (PMC9522302; doi:10.1371/journal.pone.0265160)
Supplement: Attachment — Submitted filename: Rebuttal Letter_v2.docx [file pone.0265160.s007.docx]

**Rebuttal Letter**

Journal Requirements:

When submitting your revision, we need you to address these additional requirements.

1. Please ensure that your manuscript meets PLOS ONE's style requirements, including those for file naming. The PLOS ONE style templates can be found at
   <https://journals.plos.org/plosone/s/file?id=wjVg/PLOSOne_formatting_sample_main_body.pdf> and 
   https://journals.plos.org/plosone/s/file?id=ba62/PLOSOne_formatting_sample_title_authors_affiliations.pdf.

We adjusted the formatting according to the PLOS ONE guidelines.

2. Thank you for stating the following in your Competing Interests section:
   [No]. 
   Please complete your Competing Interests on the online submission form to state any Competing Interests. If you have no competing interests, please state "The authors have declared that no competing interests exist.", as detailed online in our guide for authors at http://journals.plos.org/plosone/s/submit-now 
   This information should be included in your cover letter; we will change the online submission form on your behalf.

We changed the section “Competing Interests with the statement: “The authors have declared that no competing interest exist.”

3. In your Data Availability statement, you have not specified where the minimal data set underlying the results described in your manuscript can be found. PLOS defines a study's minimal data set as the underlying data used to reach the conclusions drawn in the manuscript and any additional data required to replicate the reported study findings in their entirety. All PLOS journals require that the minimal data set be made fully available. For more information about our data policy, please see <http://journals.plos.org/plosone/s/data-availability>.

Upon re-submitting your revised manuscript, please upload your study’s minimal underlying data set as either Supporting Information files or to a stable, public repository and include the relevant URLs, DOIs, or accession numbers within your revised cover letter. For a list of acceptable repositories, please see <http://journals.plos.org/plosone/s/data-availability#loc-recommended-repositories>. Any potentially identifying patient information must be fully anonymized.

Important: If there are ethical or legal restrictions to sharing your data publicly, please explain these restrictions in detail. Please see our guidelines for more information on what we consider unacceptable restrictions to publicly sharing data: <http://journals.plos.org/plosone/s/data-availability#loc-unacceptable-data-access-restrictions>. Note that it is not acceptable for the authors to be the sole named individuals responsible for ensuring data access.

We will update your Data Availability statement to reflect the information you provide in your cover letter.

An Excel sheet with raw data and analysis is attached as supplementary data file.

3. PLOS ONE now requires that authors provide the original uncropped and unadjusted images underlying all blot or gel results reported in a submission’s figures or Supporting Information files. This policy and the journal’s other requirements for blot/gel reporting and figure preparation are described in detail at <https://journals.plos.org/plosone/s/figures#loc-blot-and-gel-reporting-requirements> and <https://journals.plos.org/plosone/s/figures#loc-preparing-figures-from-image-files>. When you submit your revised manuscript, please ensure that your figures adhere fully to these guidelines and provide the original underlying images for all blot or gel data reported in your submission. See the following link for instructions on providing the original image data: <https://journals.plos.org/plosone/s/figures#loc-original-images-for-blots-and-gels>.
     
   In your cover letter, please note whether your blot/gel image data are in Supporting Information or posted at a public data repository, provide the repository URL if relevant, and provide specific details as to which raw blot/gel images, if any, are not available. Email us at [plosone@plos.org](mailto:plosone@plos.org) if you have any questions.

We uploaded the uncropped and unadjusted images from Western Blots as “S1_raw_images.tif”.

4. Please upload a copy of Supporting Information Figure S2 which you refer to in your text on page 27.

We attached the Figure “S2 Fig” as a .tif file.

5. Please review your reference list to ensure that it is complete and correct. If you have cited papers that have been retracted, please include the rationale for doing so in the manuscript text, or remove these references and replace them with relevant current references. Any changes to the reference list should be mentioned in the rebuttal letter that accompanies your revised manuscript. If you need to cite a retracted article, indicate the article’s retracted status in the References list and also include a citation and full reference for the retraction notice.

We are not aware of using retracted references.

Additional Editor Comments:
Please address reviewers’ questions and concerns clearly and adequately.


Reviewer's Responses to Questions

**Comments to the Author**

1. Is the manuscript technically sound, and do the data support the conclusions?

The manuscript must describe a technically sound piece of scientific research with data that supports the conclusions. Experiments must have been conducted rigorously, with appropriate controls, replication, and sample sizes. The conclusions must be drawn appropriately based on the data presented.

Reviewer #1: Yes

Reviewer #2: Yes

2. Has the statistical analysis been performed appropriately and rigorously?

Reviewer #1: Yes

Reviewer #2: Yes

3. Have the authors made all data underlying the findings in their manuscript fully available?

The [PLOS Data policy](http://www.plosone.org/static/policies.action#sharing) requires authors to make all data underlying the findings described in their manuscript fully available without restriction, with rare exception (please refer to the Data Availability Statement in the manuscript PDF file). The data should be provided as part of the manuscript or its supporting information, or deposited to a public repository. For example, in addition to summary statistics, the data points behind means, medians and variance measures should be available. If there are restrictions on publicly sharing data—e.g. participant privacy or use of data from a third party—those must be specified.

Reviewer #1: Yes

Reviewer #2: No

4. Is the manuscript presented in an intelligible fashion and written in standard English?

PLOS ONE does not copyedit accepted manuscripts, so the language in submitted articles must be clear, correct, and unambiguous. Any typographical or grammatical errors should be corrected at revision, so please note any specific errors here.

Reviewer #1: Yes

Reviewer #2: Yes

5. Review Comments to the Author

Please use the space provided to explain your answers to the questions above. You may also include additional comments for the author, including concerns about dual publication, research ethics, or publication ethics. (Please upload your review as an attachment if it exceeds 20,000 characters)

Reviewer #1: Gimbel et al. examined the role of TUG1 long non-coding RNA in endothelial functions. TUG1 is well preserved between rodents and humans, and its expression is reduced during organisms’ aging as well as cell passaging.

The authors conducted knockdown and overexpression in HUVEC and found that TUG1 is not involved in proliferation, apoptosis, migration, barrier function, mitochondrial function, and inflammation. VEGF-induced sprouting angiogenesis is reduced by TUG1 knockdown. It is consistent in two knockdown protocols. However, TUG overexpression did not increase VEGF-induced sprouting.

The research group sought upstream regulator for TUG1 expression. Oxidative stress did not influence. VEGF-A and TNF did not change TUG1, either. Notch activator did not change TUG1, either.

The authors concluded cell- and context-specific function of TUG1 because of its significant functions in other cell types like podocytes of kidney and cancer cells.

Well written methodology and results. Interpretation of the data is reasonable. The conclusion is supported by the data. I would suggest further discussion of the study limitation in comparison with previous studies demonstrated positive results.

The functional studies in this study rely on the short-term transduction —24 h transfection and 3 day maintenance. If we compare to the method in the paper (ref. 6), the use of short-term transduction may be a limitation. In this paper, stable clones were generated using CRISPR/Cas9 system including knockout and overexpression. Also, in vivo overexpression was performed to demonstrate the overexpression rescue TUG1-dependent phenotype in vivo diabetic milieu of db/db mice. Importance of in vivo model is suggested by the negative results that in vitro stimulation and senescence doesn’t reproduce TUG1 reduction, which is shown in vivo aging condition.

Another limitation may be lack of the use of high glucose condition. PGC-1 and mitochondria biogenesis is downstream target of TUG1 by ref. 6. Although knockdown in normal glucose showed significant changes in podocytes in this paper. High glucose culture (25 mM glucose) was used to test the effect of TUG1 overexpression. This suggests that TUG1 may be significant in metabolically challenged conditions in high glucose, acidic environment and hypoxia.
By the way, glucose concentration is not disclosed. It should be written.

We thank the reviewer for the suggestions to improve our manuscript. We adapted the discussion session and now included a paragraph on the limitations of the study based on the comments from reviewer #1:

We discussed the use of short-term transfection in HUVECs and its benefits compared to CRISPR/Cas system.

We also discussed the reason for usage of assays only *in vitro* based on recently published data on TUG1^-/-^ mice lacking any phenotype under basal conditions except for sperm deformation in male mice.

The glucose concentration of the EBM medium was mentioned in the Discussion part and brought into context (page 30).

Reviewer #2: Summary: The current research article by Gimbel et al presents an interesting account on the role of Taurine upregulated Gene 1 (Tug1) in Endothelial cells (ECs) in context to aging-induced cardiovascular diseases. They demonstrated peculiar expression pattern of Tug1 aging endothelial cells compared to young mouse and/or early passage of human ECs. However, the silencing of Tug1 has no effect on the homeostatic EC function. This is very interesting that, the attenuation of Tug1 in aged human and mouse ECs is dispensable for EC function.
Strengths of the work: Projected evidence in favor of Tug1 expression in young vs aged mouse and human ECs (early vs late passage). Secondly the authors are providing the strong data in terms of homeostatic function of Tug1 in ECs by studying various functional parameters like proliferation, apoptosis, barrier function, migration, mitochondrial function, and inflammatory function. Authors present strong evidence in relation to the contribution of Tug1 with VEGF challenge in inducing endothelial cell sprouting, the effect is minimal, though it is significant.
Overall: In my opinion, the manuscript is interesting, and the experiments appear carefully conducted using both in vivo and in vitro approach. I have few concerns.
• Does VEGF challenge decreases EC migration along with sprouting in Tug1 silenced ECs? If so, how this is relevant to aging-induced CVD, discuss.
• What are the differential feature counts of LNA ctrl vs LNA TUG1 from bulk RNA seq data?
• There is no mention of data availability in repository.

We thank the reviewer for these insightful suggestions for improving the manuscript.

We performed the ECIS experiment combining the effects of LNA GapmeR-mediated TUG1 knockdown and VEGFA stimulation. We could not detect any changes comparing Ctrl vs. TUG1 knockdown conditions (discussed on page 28; S3 Fig).

We added the differential feature counts in the manuscript on page 22.

We added the raw data in the Supplementary information.

6. PLOS authors have the option to publish the peer review history of their article ([what does this mean?](https://journals.plos.org/plosone/s/editorial-and-peer-review-process#loc-peer-review-history)). If published, this will include your full peer review and any attached files.


If you choose “no”, your identity will remain anonymous but your review may still be made public.


**Do you want your identity to be public for this peer review?** For information about this choice, including consent withdrawal, please see our [Privacy Policy](https://www.plos.org/privacy-policy).

Reviewer #1: No

Reviewer #2: No
